# Supplementary material for: The Inhibitory Effect of Hedera helix and Coptidis Rhizome Mixture in the Pathogenesis of Laryngopharyngeal Reflux: Cleavage of E-Cadherin in Acid-Exposed Primary Human Pharyngeal Epithelial Cells
Source: Int J Mol Sci. 2024 Nov 14;25(22):12244. doi: 10.3390/ijms252212244 (PMC11595113; doi:10.3390/ijms252212244)
Supplement: Supplementary file 1 [file ijms-25-12244-s001.zip › ijms-3296838-supplementary.pdf]

Supplementary Material

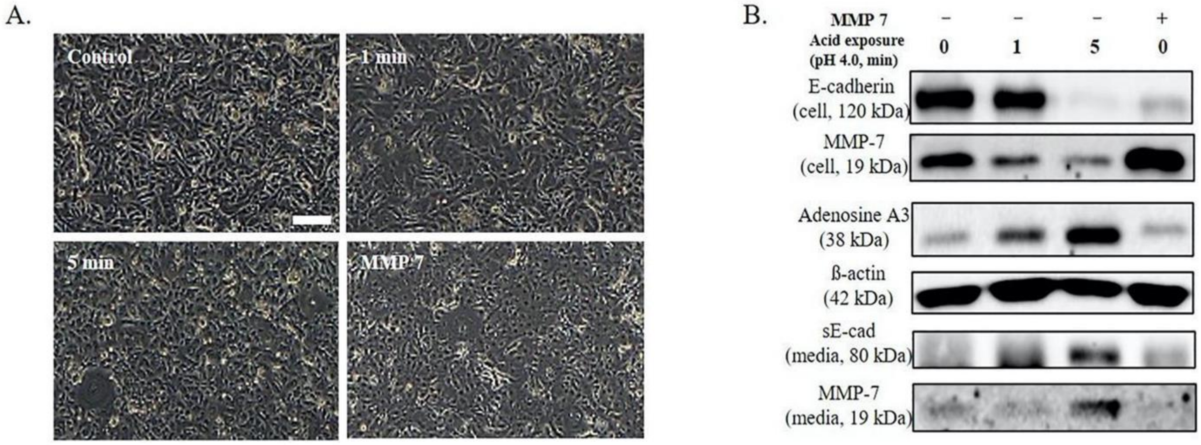

Figure S1. Changes in adenosine A3 expression according to the E-cadherin cleavage in a non-acidic environment. (A) Morphology of pharyngeal epithelial cells after acid exposure or MMP-7 treatment. Scale bar = 200 µm. (B) In acidic environments, adenosine A3 expression increased in proportion to E-cadherin cleavage, whereas no change was observed in non-acidic environments.
